# Mitigating heat stress impact on laying quails: antioxidant effects of grape seed and black mulberry leaf powder on growth, egg quality, and biochemical parameters

**DOI:** 10.1007/s11250-026-04868-3

**Published:** 2026-01-16

**Authors:** Aslıhan Sur, Muhittin Zengin, Oğuz Koray Bacaksiz, Ergün Demir, Selim Esen

**Affiliations:** 1 Kepsut Vocational School, Balıkesir University, TR Balıkesir, 10660 Turkey; 2 Department of Animal Nutrition and Nutritional Disease, Faculty of Veterinary Medicine, Balıkesir University, TR Balıkesir, 10145 Turkey; 3 Department of Animal and Dairy Sciences, University of Wisconsin, Madison, 53706 USA; 4 Republic of Türkiye Ministry of Agriculture and Forestry, Balikesir Directorate of Provincial Agriculture and Forestry, Balıkesir, TR 10470 Turkey

**Keywords:** Heat stress, Laying quail, Grape seed, Black mulberry leaf, Egg quality

## Abstract

Heat stress (HS) has a significant effect on poultry husbandry, especially laying breed, which lowers productivity and egg quality. In this study, grape seed powder (GSP) and black mulberry leaf powder (BMLP) were evaluated as natural antioxidants against HS effects on laying quails. The trial involved 288 8-week-old laying quails in a 2 × 3 factorial design with (six replicates and eight quails in each) two temperatures (thermoneutral and HS) and three diets (Control, 3%GSP, 4%BMLP). Feed intake was shown to be negatively impacted by HS, however its effects were mitigated by GSP and BMLP (*P <* 0.05). Despite HS-induced reductions in egg and shell weight, BMLP significantly prevented egg weight loss (*P* < 0.05). A positive impact of GSP and BMLP could be seen on egg weight, albumen quality, and yolk color (*P* < 0.05). Serum biochemistry analysis indicated that GSP and BMLP reduced cholesterol and triglyceride levels, while BMLP showed superiority in decreasing malondialdehyde (MDA), an oxidative stress marker (*P* < 0.05). Furthermore, both additives improved albumen quality and reduced oxidative stress. These findings emphasize the potential of natural antioxidants, particularly BMLP, for enhancing quail resilience to HS and maintaining overall health. Therefore, incorporating GSP and BMLP into quail diets is recommended to mitigate the detrimental effects of HS on productivity and egg quality.

## Introduction

Layer farming, as an integral part of the global livestock industry, faces many problems that adversely affect bird welfare, physiological status, and productivity. One of the most common problems that poultry farms face, especially laying breeding, is heat stress (**HS**) (El-hack et al. 2017). Heat stress, in general, can increase the mobilization and excretion of vitamins and minerals from tissues, more than compensating for any minor initial deficiencies resulting in poor production performance (Sahin et al. 2008). Research has shown that high environmental temperatures cause oxidative stress in birds, weaken the antioxidant defence system, and cause poultry to produce less (Omid et al. 2018; İflazoğlu Mutlu and Güler 2021). Furthermore, previous research has linked HS with reduced feed intake (**FI**) and feed efficiency, leading to decreased ovarian weight and number of follicles and overall egg production and poorer egg quality (eggs that are lighter, have a smaller yolk and have a lower percentage or weight of yolk) (El-tarabany 2016; Pu et al. 2018). Therefore, it becomes increasingly important to address the adverse effects of HS on quail populations in the long term with the ongoing rise in global temperatures attributed to climate change.

Alterations in diet and management practices can reduce the adverse effect of HS on laying hen production performance, feed efficiency, and egg quality. In parallel with increasing consumer awareness and concerns, low-cost synthetic antioxidants, which have been used effectively in recent years, have begun to be replaced by natural antioxidants (Ao and Kim 2016; Turcu et al. 2021).

One such source of natural antioxidants is grape seed. Due to its high phenolic and flavonoid content, grape seed powder (Sur et al. 2023), meal (Nan et al. 2022) has been widely examined for utilization in broiler and laying hen diets. Prior studies have also demonstrated that grape seed flavonoids increase the number of eggs laid by stimulating follicular maturation and ovulation (Sun et al. 2018; Hafeez et al. 2023). Furthermore, Kara et al. (2016) found that feeding grape pomace flour to laying hens significantly reduced lipid peroxidation and significantly increased egg antioxidant capacity, while Hafeez et al. (2023) demonstrated that grape seed extract significantly increased egg weight, egg shell weight, and egg shell thickness.

Research reveals that mulberry leaves are promising for use in poultry diets due to their high protein, Ca, and P content, richness in essential amino acids lysine and leucine, and almost zero antinutritional compounds (Al-kirshi et al. 2013; Ustundag and Ozdogan 2023). The inclusion of mulberry leaves in the diet has been found to increase eggshell weight and shell strength while decreasing the feed conversion ratio (FCR), which may be related to an improved oxidative status of birds (Lin et al. 2016). Furthermore, *Bacteroidetes* members, which are known to play a significant role in digesting typically indigestible polysaccharides from the diet through fermentation, were observed to increase in chickens fed diets containing mulberry leaf powder in a microbiota investigation (Chen et al. 2019). It has also been suggested that the xanthophylls found in mulberry leaves alter the color of egg yolks by turning them to a darker red (Lokaewmanee et al. 2009).

Although grape seed powder (GSP) and black mulberry leaf powder (BMLP) have been studied separately in broilers (Zhang et al. 2022.; Chen et al. 2019; Israr et al. 2021; Qin et al. 2022) and quails (Erişir et al. 2018; Sur et al. 2023; Ustundag and Ozdogan 2023), the function of GSP and BMLP in laying quails remains mostly unknown. As a result, the goal of this study was to evaluate GSP and BMLP (which are known for their antioxidant characteristics) growth, egg quality, antioxidan and biochemical parameters on laying quails exposed to HS.

## Materials and methods

### Ethical approval

The Ethics Committee of Balikesir University approved the research protocol before the study began, and the Declaration of Helsinki and this research protocol were strictly followed throughout the trial.

### Animals, diets and experimental design

The research animal material consists of 288 8-week-old laying quails (*Coturnix coturnix japonica*) obtained from the Quail Breeding Unit of the Balıkesir University Livestock Application and Research Center Farm, Balıkesir, Türkiye. Six groups consisting of 48 female quails were weighed and randomly assign into 6 replicates and each replicate included 8 quails. The research was conducted in a 2 × 3 factorial design involving two temperatures (thermoneutral and high temperature) and three diets (0%, 3%GSP, and 0%, 4% BMLP).

Each replicate was housed in cages 40 cm long and 32 cm wide with a density of 160 cm^2^/quail. The doses of GSP and BMLP used in this study were determined according to Brenes et al. (2008) and Chen et al. (2019) respectively. Although different doses were used in the studies, we aimed to investigate the effects of 3% (GSP) and 4% (BMLP) doses.The ingredient and nutrient composition of the diets are presented in Table 1. Corn and soybean meal-based feeds were formulated to be isonitrogenic and isoenergic according to the National Research Council (1994) recommendation. GSP were purchased from a Turkish factory (Lermonos) in Denizli. Black mulberry leaves were collected from black mulberry trees in Turkey and then dried and turned into powder. The additives (BMLP and GSP) were homogeneously mixed carefully to the basal diet. The powder form basal diet prepared according to National Research Center was supplied by a commercial company and spilled feeds are calculated.

**Table 1:** Ingredients and chemical composition of basal diet

Ingredients	Control	GSP	BMLP
Corn Grain, yellow	57	57	57
Soybean meal, 44% CP	28	28.1	28
Wheat bran	4	3.6	3.6
Soybean Oil	3.3	3.3	3.3
GSP	-	0.3	-
BMLP	-	-	0.4
Dicalcium phosphate	1.3	1.3	1.3
DL-Methionine	0.16	0.16	0.16
Lysine	0.10	0.10	0.10
Limestone	5.5	5.5	5.5
Sodium chloride	0.35	0.35	0.35
Vitamin premix^c^	0.30	0.30	0.30
Mineral premix^d^	0.30	0.30	0.30
**Nutritional composition**			
CP, g/kg	179.5	179.6	179.6
EE, g/kg	5.07	5.07	5.08
Ash, g/kg	9.95	9.95	9.98
CF, g/kg	3.62	3.74	3.67
Ca, g/kg	2.50	2.50	2.50
P ^b^, g/kg	0.350	0.342	0.342
ME kcal/kg ^a^, DM	2929.3	2930.2	2931.4
TP, mg/kg	-	9.04	4.54

GSP^e^: grape seed powder, BMLP^f^: black mulberry leaf powder, ME: metabolizable energy, DM: dry matter, CP: crude protein, EE: ether extract, CF: crude fiber; TP: total phenolic (given as gallic acid equivalent)

^**a**^ Analyzed

^**b**^ Available

^**c**^ A vitamin premix contains 10.000 IU of vitamin A (retinyl acetate); 2.500 IU of vitamin D3 (cholecalciferol); 40 mg of vitamin E (alphatocopherol); 4 mg of vitamin K3 (menadione sodium bi-sulfte); 3 mg of vitamin B1 (thiamine mononitrate); 6 mg of vitamin B2 (ribofavin); 25 mg of niacin (nicotinic acid); 6 mg of vitamin B6 (pyridoxine hydrochloride); 0.02 mg of vitamin B12 (cyanocobalamin); 10 mg of pantothenic acid (calcium d-pantothenate); 1 mg of folic acid; 1 mg; 0.05 mg d-biotin, 0.05

^**d**^ A mineral premix contains 80 mg of Mn; 60 mg Fe; 60 mg of Zn; 5 mg of Cu; 0.25 mg Co; 1 mg of I; 0.1 mg of Se

^e^ Analyzed (İngredients: **Phenolic acid** ((Gallic acid (331 mg/l), Caffeic acid (1.16 mg/l)), **Flavonoids** ((Catechin (4.10 µg/g), Naringin (3.40 µg/g), Rutin (1.80 µg/g), Myricetin (0.75 µg/g), Morin (0.23 µg/g), Naringenin (2 µg/g), Quercetin (4 µg/g), **Stilbenes** ((Resveratrol (0.50 µg/g)

^f^ Analyzed (Ingredients; ((**Phenolic acid** ((Protocatechuic acid (8.70 mg GAE/g), Chlorogenic acid (587.39 mg GAE/g), t-Cinnamic acid(4.79 mg GAE/g)), **Flavonoids** ((Rutin (47.01 mg QUE/g), Pinocembrin (1.70 mg QUE/g)) **(Kolaylı et al.**^2024^**)**

Each group had its own controlled environment, where the temperature and relative humidity (**RH**) could be adjusted to their specifications. A temperature and humidity setting of 22 °C and 58% RH was maintained for the thermoneutral groups during the trial period, and 34 °C and 50% RH for the heat stressed groups between 9:00 am and 7:00 pm, respectively, and 22 °C and 58% RH between 7 pm and 9 am. In both the thermoneutral and heat stressed groups, the lighting schedule was 16 h light: 8 h dark with *ad libitum* feeding and drinking for the course of the trial.

### Performance variables and egg quality

As part of the trial, each quail was weighed at the start and end of it, and its weight was recorded. A daily live weight gain was calculated by dividing the difference in live weight at the beginning and end of the trial by the number of days. A daily feed was given to each group, and at the end of the experiment, daily feeding was calculated by dividing the feed consumed by each group by the number of days and the number of animals in the group.

Fourty eight eggs per replicate were randomly sampled on two consecutive days per week during the last two weeks of the study, and indexes (shape index, albumen index, yolk index, and Haugh unit) and parameters measuring the external (egg weight, egg width, egg length, shell weight and shell thickness) and internal (albumen length, albumen width, albumen height, yolk height, yolk diameter and yolk color) quality of the eggs were computed. The weight of the eggs and eggshells was determined using an electronic balance (ABS 220–4 N, KERN & Sohn GmbH, Germany) with an accuracy of ± 0.01 g. A calibrated electronic vernier caliper made of stainless steel was used to measure the egg width, egg length, albumen length, albumen width, albumen height, yolk height, and yolk diameter in millimeters, with a precision of ± 0.001 mm. The weight and thickness of each eggshell were measured after it was carefully broken open and separated and dried at 105 °C overnight. Once the external characteristics of the eggs had been determined, the contents of the eggs were transferred to petri dish for subsequent quality measurements. The weight of the yolk was initially ascertained through a gently separating albumen from the yolk. The albumen weight was then determined by subtracting the weights of the shell and yolk from the egg. The yolk color was scored using Hoffman-La Roche yolk colour fans (Vuilleumier 1969). Shape index, albumen index, and yolk index were calculated as described by Mehaisen et al. (2019) and expressed as percentages (%). The Haugh unit, on the other hand, was calculated using the method described by Ryu et al. (2011).

### Serum biochemical and oxidative stress parameters

During the final day of the trial (on day 42), the quails were given only clean water and no feed. Finally, a quail was selected at random from each replicate and decapitated to collect blood in EDTA tubes. For biochemical analysis, the blood samples were centrifuged at 3000 g for 10 min. Then, the upper layer was transferred to clean plastic microtubes and stored at -20 °C. Serum glucose, total protein, albumin, globulin, urea, blood urea nitrogen (**BUN**), creatinine, cholesterol, triglycerides, calcium, phosphorus, alanine aminotransferase (**ALT**), and aspartate aminotransferase (**AST**) were measured using a Mindray BS400 (Shenzhen, China) autoanalyzer, and the BUN/creatinine and albumin/globulin ratios were calculated.

Total antioxidant status (**TAS**) and total oxidant status (**TOS**) levels were determined following the automatic measurement method of Rel Assay (Rel Assay Diagnostics Kit, Mega Tip, Gaziantep, Turkey) procedure developed by Erel (2004). The ratio of TOS to TAS, known as the oxidative stress index (**OSI**), was then calculated. Following the method outlined by Israr et al. (2021), the samples’ malondialdehyde (**MDA**) levels were determined spectrophotometrically at 532 nm with thiobarbituric acid. The levels of cortisol and tumour necrosis factor-alpha (**TNF-α**) in the samples were determined utilizing an ELISA reader (Biotek ELx800; BioTek Instruments, Inc., Vermont, USA) along with commercial ELISA kits (BT-LABS, Shanghai Crystal Day Biotech Co., Ltd.).

### Statistical analysis

The experimental design of the study was completely randomized. The significance levels (p) and standard errors of the experimental findings are presented in the tables. Statistical analyses were performed using the General Linear Model in the SPSS software package with a 2 × 3 experimental design to examine the main effects and interactions of temperature (thermoneutral (N) and heat stress (H)) and diet (control, GSP, BMLP). Duncan’s multiple comparison test was performed when the interaction showed a statistically significant difference.

## Results

### Growth performance of quails

Daily feed intake was shown to be significantly affected by temperature and diet (*P* ˂ 0.0001) and by the interaction between the two (*P* = 0.0003), but there was no effect of diet, temperature or the diet × temperature interaction on final body weight (Table 2). Even though groups that were subjected to HS experienced a decrease in feed consumption, both GSP and BMLP were successful in avoiding the decrease in feed consumption. Although HS reduced feed consumption by 36.67% in the CH group, it dropped to 18.41% in the GSPH group and 1.09% in the BMLPH group. However, diets that improve daily feed intake do not have the same effect on daily weight growth. Those in the BMLP-fed groups (BMLPN and BMLPH) lose weight compared to the control groups whose diets have the highest daily weight gain (*P* = 0.0223).

**Table 2:** Effects of grape seed and black mulberry leaf powder on performance parameters under heat stress conditions

AT	AG	Initial Body Weight (g)	Final Body Weight (g)	Daily Feed Intake (g)	Daily Weight Gain (g)
T	C	204.71 ± 16.92	203.83 ± 47.81	19.28 ± 3.72	0.21 ± 0.03
	BMLP	208.00 ± 23.14	204.10 ± 26.44	20.89 ± 3.50	-0.09 ± 0,01
	GSP	209.39 ± 19.87	207.60 ± 36.32	17.80 ± 2.06	0.07 ± 0.02
SEM		1.61	3.04	19.79	1.95
HS	C	210.00 ± 22.05	203.87 ± 46.02	12.21 ± 3.01	0.07 ± 0.02
	BMLP	211.21 ± 16.72	207.08 ± 17.48	19.07 ± 2.34	-0.015 ± 0,01
	GSP	207.31 ± 16.12	203.64 ± 35.64	15.79 ± 2.16	0.01 ± 0.03
SEM		1.61	3.04	21.38 ± 4.45	2.76
P(D)		0.8673	0.2167	< 0.0001**	0.0223*
P(T)		0.5379	0.7571	< 0.0001**	0.2581
P(D*T)		0.6578	0.8612	0.0003*	0.8369

AT: Ambient Temperature, AG: Application Groups, T: Thermoneutral Conditions, HS: Heat Stress Conditions, C: Quails fed a basal diet – Control, BMLP: Quails fed a %4 Black Mulberry Leaf Powder Diet, GSP: Quails fed a %3 Grape Seed Powder Diet, SEM: Standard Error Mean, (*P < 0.05, **P < 0.001), D: Diet, T: Temperature, D*T: Diet x Temperature Interaction

### Egg quality of quails

An overview of external egg quality traits of quails can be found in Table 3. Heat stress-exposed groups exhibited a reduction in egg weight, length, and width (*P* < 0.0001). Except for the control group, similar findings emerged for shell weight (*P* = 0.0209). In HS-exposed groups, diet might attenuate the decline in egg weight and eggshell (*P* < 0.0001). In the BMLP-treated group, it superior to GSP in terms of preventing egg weight and eggshell loss. Further, shell weight (*P* = 0.0299) and egg weight (*P* = 0.0087) were both significantly affected by the temperature × diet interaction.

**Table 3:** Effects of grape seed and black mulberry leaf powder on external egg quality parameters under heat stress conditions

AT	AG	Egg Weight (g)	Egg Length (mm)	Egg Width (mm)	Shell Weight (g)	Shell Thickness (mm)
T	C	11.05 ± 0.75	31.77 ± 1.27	24.92 ± 0.68	0.98 ± 0.08	0.32 ± 0.04
	BMLP	11.31 ± 0.80	32.79 ± 1.33	24.94 ± 0.73	1.03 ± 0.08	0.35 ± 0.05
	GSP	11.13 ± 0.60	31.90 ± 1.47	25.21 ± 1.67	1.04 ± 0.16	0.34 ± 0.05
SEM		0.066	0.106	0.081	0.010	0.003
HS	C	9.86 ± 0.87	30.40 ± 1.16	24.11 ± 0.78	1.00 ± 0.15	0.32 ± 0.04
	BMLP	10.82 ± 0.77	32.25 ± 1.21	24.56 ± 0.57	0.97 ± 0.10	0.35 ± 0.04
	GSP	10.25 ± 0.93	30.98 ± 1.18	24.42 ± 0.89	0.98 ± 0.09	0.33 ± 0.03
SEM		0.066	0.106	0.081	0.010	0.003
P(D)		< 0.0001**	< 0.0001**	0.0987	0.5584	0.8389
P(T)		< 0.0001**	< 0.0001**	< 0.0001**	0.0203*	0.5920
P(D*T)		0.0087*	0.0724	0.2018	0.0299*	0.3846

AT: Ambient Temperature, AG: Application Groups, T: Thermoneutral Conditions, HS: Heat Stress Conditions, C: Quails fed a basal diet – Control, BMLP: Quails fed a %4 Black Mulberry Leaf Powder Diet, GSP: Quails fed a %3 Grape Seed Powder Diet, SEM: Standard Error Mean, (*P < 0.05, **P < 0.001), D: Diet, T: Temperature, D*T: Diet x Temperature Interaction

In Table 4, internal egg quality traits of quails are shown. Diet and temperature had a substantial effect on yolk height and diameter (*P* < 0.0001), however the interaction between the two was insignificant. Albumen length was also significantly affected by temperature (*P* < 0.0001) and diet (*P* = 0.0110), but there was no significant interaction between the two. Albumen height, on the other hand, was significantly reduced by diet (*P* < 0.0001), temperature (*P* = 0.0022), and the diet × temperature interaction (*P* = 0.0423). However, this reduction was less pronounced in diets containing GSP and BMLP. Furthermore, temperature had a significant effect on yolk color (*P* = 0.0013), while diet did not.

**Table 4:** Effects of grape seed and black mulberry leaf powder on internal egg quality parameters under heat stress conditions

AT	AG	Albumen Length (mm)	Albumen Height (mm)	Albumen Width (mm)	Yolk Height (mm)	Yolk Diameter (mm)	Yolk Color (Points)
T	C	75.18 ± 8.85	2.82 ± 0.45	66.60 ± 7.09	8.74 ± 0.85	20.01 ± 0.87	11.40 ± 0.78
	BMLP	77.17 ± 8.67	3.16 ± 0.41	64.50 ± 8.84	9.33 ± 0.58	22.30 ± 1.75	11.16 ± 0.90
	GSP	73.10 ± 6.76	2.92 ± 0.71	64.41 ± 6.40	8.70 ± 0.83	20.44 ± 1.44	11.41 ± 0.63
SEM		0.598	0.031	0.531	0.054	0.086	0.058
HS	C	71.44 ± 9.33	2.43 ± 0.48	61.13 ± 6.36	8.17 ± 0.47	18.10 ± 1.44	10.77 ± 0.78
	BMLP	72.68 ± 7.69	3.12 ± 0.42	63.86 ± 6.71	8.85 ± 0.78	20.01 ± 0.98	11.12 ± 0.91
	GSP	70.38 ± 6.47	2.77 ± 0.51	60.01 ± 6.43	8.19 ± 0.68	19.66 ± 0.77	11.04 ± 0.80
SEM		0.598	0.031	0.531	0.054	0.086	0.058
P(D)		0.0110*	< 0.0001**	0.0940	< 0.0001**	< 0.0001**	0.4437
P(T)		< 0.0001**	0.0022*	< 0.0001**	< 0.0001**	< 0.0001**	0.0013*
P(D*T)		0.5803	0.0423*	0.0670	0.7707	0.0855	0.0602

AT: Ambient Temperature, AG: Application Groups, T: Thermoneutral Conditions, HS: Heat Stress Conditions, C: Quails fed a basal diet – Control, BMLP: Quails fed a %4 Black Mulberry Leaf Powder Diet, GSP: Quails fed a %3 Grape Seed Powder Diet, SEM: Standard Error Mean, (*P < 0.05, **P < 0.001), D: Diet, T: Temperature, D*T: Diet x Temperature Interaction

Table 5 shows the external and internal shape indexes of quail eggs, as well as data from the Haugh unit. The diet had a significant impact on the shape index, albumen index, yolk index, and Haugh unit (*P* < 0.0001); however, temperature had a significant effect only on the yolk index (*P* = 0.0015). The albumen index and Haugh unit values of eggs laid by quails given BMLP-containing diets were higher compared to those laid by quails given control or GSP diets, while the Haugh unit values were lower (*P* < 0.0001).

**Table 5:** Effects of grape seed and black mulberry leaf powder on some egg indexes and Haugh unit under heat stress conditions

AT	AG	Shape Index (%)	Albumen Index (%)	Yolk Index (%)	Haugh Unit
T	C	78.55 ± 2.98	4.03 ± 0.86	49.72 ± 4.23	79.52 ± 3.24
	BMLP	76.11 ± 3.04	4.49 ± 0.69	42.04 ± 3.47	81.60 ± 2.83
	GSP	77.80 ± 2.76	4.27 ± 1.09	42.61 ± 3.55	79.92 ± 3.60
SEM		0.232	0.058	0.284	0.214
HS	C	79.35 ± 2.46	3.69 ± 0.75	45.35 ± 3.32	77.97 ± 3.69
	BMLP	76.26 ± 2.79	4.62 ± 0.86	44.28 ± 3.89	81.77 ± 2.83
	GSP	78.89 ± 3.04	4.28 ± 0.85	42.94 ± 3.97	79.99 ± 3.32
SEM		0.232	0.058	0.284	0.214
P(D)		< 0.0001**	< 0.0001**	0.0096*	< 0.0001**
P(T)		0.6795	0.6084	0.0015*	0.3484
P(D*T)		0.5244	0.1009	0.1617	0.1614

AT: Ambient Temperature, AG: Application Groups, T: Thermoneutral Conditions, HS: Heat Stress Conditions, C: Quails fed a basal diet – Control, BMLP: Quails fed a %4 Black Mulberry Leaf Powder Diet, GSP: Quails fed a %3 Grape Seed Powder Diet, SEM: Standard Error Mean, (*P < 0.05, **P < 0.001), D: Diet, T: Temperature, D*T: Diet x Temperature Interaction

### Serum biochemistry and antioxidant status of quails

In the current study, diet had a significant effect on urea (*P* = 0.0421), BUN (*P* = 0.0421), cholesterol (*P* = 0.0005), calcium (*P* = 0.0006), ALT (*P* = 0.0033), and AST (*P* = 0.0304) levels and albumin/globulin ratio (*P* = 0.0047), whereas calcium (*P* = 0.0032) and AST (*P* = 0.0011) levels were significantly affected by temperature (Table 6). Further, the diet and temperature did not show any significant interaction. Diets containing BMLP had a higher albumin/globulin ratio than control and GSP diets, but lower values for urea, BUN, cholesterol, triglycerides, calcium, ALT, and AST.

**Table 6:** Effects of grape seed and black mulberry leaf powder on some serum biochemical parameters under heat stress conditions

AT	AG	GLU (mg/dl)	TP (g/dl)	ALB (g/dl)	GLO (mg/dl)	UREA (mg/dl)	BUN (mg/dl)	CRE (mg/dl)	CHO (mg/dl)	TRI (mg/dl)	CAL (mg/dl)	PHO (mg/dl)	ALT (U/I)	AST (U/I)	BUN/CRE	ALB/GLO
T	C	321.30 ± 18.34	3.57 ± 0.64	1.33 ± 0.22	2.33 ± 0.42	26.70 ± 13.21	12.47 ± 6.17	0.27 ± 0.11	215.17 ± 52.74	1377.30 ± 407.00	21.77 ± 4.01	6.69 ± 1.99	13.25 ± 13.36	441.00 ± 107.35	81.27 ± 107.43	0.60 ± 0.03
	BMLP	323.32 ± 38.14	3.37 ± 0.34	1.33 ± 0.20	2.03 ± 0.17	12.85 ± 15.45	6.00 ± 7.22	0.30 ± 0.03	126.33 ± 20.21	818.87 ± 324.99	21.53 ± 3.60	5.80 ± 1.10	4.78 ± 1.68	408.00 ± 70.41	19.27 ± 21.92	0.65 ± 0.07
	GSP	327.60 ± 35.56	4.08 ± 0.75	1.43 ± 0.19	2.65 ± 0.59	26.60 ± 13.29	12.42 ± 6.21	0.21 ± 0.10	278.17 ± 131.70	1701.78 ± 711.99	25.16 ± 7.58	8.36 ± 2.49	17.28 ± 8.28	540.33 ± 128.61	94.99 ± 102.34	0.44 ± 0.07
SEM		9.59	0.19	0.04	0.17	3.54	1.65	0.01	17.00	108.82	1.21	0.44	1.66	19.79	15.09	0.02
HS	C	310.55 ± 57.17	4.22 ± 1.42	1.25 ± 0.10	2.97 ± 1.49	28.27 ± 17.11	13.20 ± 7.99	0.32 ± 0.06	230.50 ± 35.00	1233.98 ± 377.83	21.34 ± 4.21	6.58 ± 1.74	11.22 ± 3.92	342.17 ± 49.38	42.43 ± 24.24	0.48 ± 0.15
	BMLP	359.70 ± 35.10	3.13 ± 0.49	1.27 ± 0.16	1.87 ± 0.34	12.08 ± 17.80	5.64 ± 8.31	0.31 ± 0.02	101.50 ± 30.39	576.30 ± 256.67	8.33 ± 1.67	5.11 ± 1.37	2.33 ± 0.75	349.67 ± 10.07	18.36 ± 26.79	0.68 ± 0.05
	GSP	319.68 ± 48.68	3.73 ± 0.77	1.37 ± 0.16	2.37 ± 0.62	24.27 ± 12.41	11.33 ± 5.79	0.27 ± 0.08	187.17 ± 92.41	1249.05 ± 540.36	22.39 ± 7.12	6.25 ± 2.16	9.40 ± 5.66	395.17 ± 82.15	46.35 ± 28.09	0.60 ± 0.10
SEM		9.59	0.19	0.04	0.17	3.54	1.65	0.01	17.00	108.82	1.21	0.44	1.66	19.79	15.09	0.02
P(D)		0.3012	0.0928	0.2648	0.0821	0.0421*	0.0421*	0.1005	0.005*	0.007*	0.0006*	0.0638	0.0033*	0.0304*	0.1224	0.0047*
P(T)		0.6662	0.9350	0.2309	0.7041	0.9194	0.9195	0.0911	0.1738	0.0793	0.0032*	0.1307	0.0894	0.0011*	0.1775	0.6445
P(D*T)		0.2963	0.2704	0.9912	0.1997	0.9502	0.9501	0.6672	0.2068	0.7066	0.0109	0.4152	0.5324	0.4573	0.6325	0.0646

AT: Ambient Temperature, AG: Application Groups, T:Thermoneutral Conditions, HS: Heat Stress Conditions, C: Quails fed a basal diet – Control, BMLP: Quails fed a %4 Black Mulberry Leaf Powder Diet, GSP: Quails fed a %3 Grape Seed Powder Diet, SEM: Standard Error Mean, (*P < 0.05, **P < 0.001), D: Diet, T: Temperature, D*T: Diet x Temperature Interaction, GLU: Glucose, TP: Total Protein, ALB: Albumin, GLO: Globulin, CRE: Creatinine, CHI: Cholesterol, TRI: Triglyceride, CAL: Calcium, PHO: Phosphorus

Table 7 shows the effects of GPS and BMLP on oxidative stress parameters in quails under HS. Although diet significantly affected MDA levels (*P* < 0.0001), neither temperature nor diet significantly affected Tnf-α, cortisol, TAS, TOS, or OSI. A lower level of MDA was detected in quail fed BMLP-containing diets.

**Table 7:** Effects of grape seed and black mulberry leaf powder on some antioxidant parameters under heat stress conditions

AT	AG	TNF-α	COR	MDA	TAS	TOS	OSI
T	C	28.07 ± 10.66	38.96 ± 12.42	56.64 ± 18.36	1.80 ± 0.24	12.26 ± 3.07	0.68 ± 0.12
	BMLP	23.31 ± 10.01	34.86 ± 13.69	24.91 ± 12.24	1.88 ± 0.20	15.19 ± 6.04	0.81 ± 0.32
	GSP	29.49 ± 14.88	40.16 ± 13.59	62.06 ± 19.89	1.91 ± 0.37	13.30 ± 3.88	0.70 ± 0.16
SEM		2.05	3.93	3.76	0.06	1.24	0.07
HS	C	24.53 ± 5.45	47.63 ± 37.40	54.57 ± 18.17	1.90 ± 0.35	16.63 ± 11.71	0.90 ± 0.66
	BMLP	28.62 ± 9.60	33.42 ± 7.71	29.03 ± 16.38	1.85 ± 0.29	10.10 ± 4.14	0.56 ± 0.25
	GSP	31.01 ± 7.73	42.97 ± 15.53	49.27 ± 23.56	1.76 ± 0.16	10.27 ± 2.54	0.58 ± 0.14
SEM		2.05	3.93	3.76	0.06	1.24	0.07
P(D)		0.4156	0.3691	< 0.0001**	0.9576	0.4563	0.4334
P(T)		0.7075	0.5504	0.5042	0.7107	0.4793	0.6256
P(D*T)		0.4658	0.7583	0.4297	0.4249	0.0807	0.1363

AT: Ambient Temperature, AG: Application Groups, T: Thermoneutral Conditions, HS: Heat Stress Conditions, C: Quails fed a basal diet – Control, BMLP: Quails fed a %4 Black Mulberry Leaf Powder Diet, GSP: Quails fed a %3 Grape Seed Powder Diet, SEM: Standard Error Mean, (*P < 0.05, **P < 0.001), D: Diet, T: Temperature, D*T: Diet x Temperature Interaction, TNF-α: Tumor necrosis Factor-Alpha, MDA: malondialdehyde, TAS: Total Antioxidant Status, TOS: Total Oxidant Status, OSI: Oxidative Stress Index

## Discussion

Prior research has shown that decreased laying performance can be attributed, in part, to HS caused by the accumulation of heat within the cages, which is especially prevalent in hot climates (Ratriyanto et al. 2020). Further, HS reduces the activity of the carbonic anhydrase enzyme in the mucosa of the shell gland, affecting the conversion of dietary calcium to calcium carbonate, which is required for egg shell formation, reducing FI and limiting the availability of calcium in the blood, which is required for egg shell formation, and lead to smaller eggs with thinner eggshells, which in turn result in lower egg production (El-tarabany 2016; De Moraes et al. 2019). Increasing the tolerance to HS and reducing negative impacts on productivity can be achieved using natural products, which have recently gained prominence as a viable option. As a result, the purpose of our research is to thoroughly investigate the effects of supplementing GSP and BMLP, which are known for their bioactive components, in the diets of laying quails, with a particular emphasis on assessing their impact on growth performance, egg quality parameters, blood biochemistry, and antioxidant status.

Research has shown that HS can have negative effects on several aspects of the intestinal villus-crypt system, including reducing the surface area and height of the villus, damaging the intestinal epithelial cells, and impairing nutrient utilization in birds (Al-Fataftah and Abdelqader 2014; Yi et al. 2016). In addition, a substantial body of evidence demonstrates that HS causes oxidative stress, which is a primary factor that is associated with decreased production performance in birds (Mujahid et al. 2006; Omid et al. 2018). In the current study, quails’ feed consumption was negatively affected by HS, but when GSP or BMLP were added, this effect was greatly reduced. However, even with increased feed consumption, daily weight gain did not increase in proportion. GSP and BMLP contain polyphenols, specifically tannins, which may bind to proteins and hinder digestion, which may explain this divergence (Hafeez et al. 2023; Ustundag and Ozdogan 2023). These results emphasize the importance of assessing the presence of condensed tannins and their potential effects on protein digestion when incorporating tannin-rich natural products into laying quail diets (Romero et al. 2022). Furthermore, it has been noted that GSP and MLP have a high fiber content and various antinutritional elements (Ding et al. 2021; Costa et al. 2022). These elements can lead to a substantial reduction in nutrients retained in the digestive tract, which in turn reduces digestion time and nutrient absorption. As a result, quail performance is negatively impacted.

Previous studies have shown that laying hens and quails exposed to the adverse effects of cyclic or constant HS experience not only compromised growth performance but also reduced egg production and quality (Liao et al. 2018; Mehaisen et al. 2019). HS has been shown to reduce the release of luteinizing hormone, a reproductive performance regulator, from the hypothalamus in laying hens. This loss in reproductive efficiency is caused by a decrease in Zn levels in the serum and liver (Sahin et al. 2002), where Zn is an essential component of many enzymes and should be kept at optimal levels (Donoghue et al. 1989). Furthermore, Zn deficiency reduces egg shell weight since it is a component of the carbonic anhydrase enzyme, which provides carbonate ions during egg shell formation (Nys et al. 2001; Cruvinel et al. 2021). Furthermore, extensive studies have shown that HS exposure in quails results in the development of lighter-weight eggs (El-tarabany 2016; Soares et al. 2019). In this study, quails subjected to HS had a decrease in both egg and shell weight.

It is recognized that the visual characteristics of egg yolk and albumen, as well as the volume of air cells, are key indicators for consumers when choosing high-quality eggs (Alagawany et al. 2017). These characteristics can be reliably assessed through the examination of egg yolk color, Haugh units, and MDA levels. Further, previous study indicates that the decrease in Haugh unit and yolk index found in birds exposed to HS is most likely due to a reduction in protein synthesis and an increase in water excretion into the egg albumen (Sahin et al. 2008). This situation is closely related to the formation of free radicals during HS, which subsequently bind to the cell membrane and lead to lipid peroxidation (Karimi et al. 2015). In the current study, the incorporation of GSP and BMLP, renowned for their antioxidant and free radical scavenging properties, results in an improvement of the albumen index in quail diets exposed to HS. Kaya et al. (2014) and Zhang et al. (2022) both documented similar results.

On the other hand, the increase in albumen viscosity is closely related to the increase in Haugh units. Previous research has shown a correlation between increased albumin and increased albumen viscosity, which decreases under HS (El-tarabany 2016). In the current study, a similar Haugh unit outcome was observed between groups that had been exposed to HS and groups that were not. It is believed that the antioxidant properties of GSP and BMLP play an important role in maintaining calcium levels in the uterus as well as yolk formation in the ovaries (Lin et al. 2016).

It is well known that cholesterol and triglycerides are essential components of blood fat, and their levels indicate how much fat is absorbed and metabolized (Nan et al. 2022).This study found that introducing GSP and BMLP into the diet of laying quails can reduce serum triglyceride and cholesterol levels. The regulatory effects of the bioactive ingredients in GSP and BMLP on quail fat metabolism are probably responsible for this result, aligning with the earlier findings of Reis et al. (2019) and Ding et al. (2021). In particular, polyphenols significantly reduce 3-hydroxy-3-methylgluteryl CoA (**HMG-CoA**) to mevalonate conversion. This mechanism, which is controlled by the rate-limiting enzyme HMG-CoA reductase, in cholesterol production, has been shown to reduce plasma and egg cholesterol levels in hens (Song et al. 2017; Sun et al. 2018). On the other hand, previous studies have established the positive impact of proanthocyanidins on blood glucose level (Vislocky and Fernandez 2010), this study found that the addition of GSP or BMLP, recognized for their high proanthocyanidin content, did not effectively lower blood glucose levels.

In most cases, the immune system’s reaction to infections or stressful situations is to produce elevated serum globulin levels (Fernández-Cruz et al. 2009). In this study, the increase in globulin levels observed in laying quails exposed to HS and fed a basal diet contributed to an overall increase in total protein. Previous research has shown that grape seeds boosts immunological and anti-inflammatory responses, resulting in a decrease in globulin levels in groups using GSP (Reis et al. 2019; Hafeez et al. 2023). An analogous situation can be observed in the case of BMLP, which is recognized for its anti-inflammatory properties.

Previous researches indicates that lower levels of AST and ALT generally signify a healthier status, while elevated levels suggest detrimental effects on liver tissues attributed to excessive stress (Zengin et al. 2022). The cyclic nature of the applied HS may explain why the HS-exposed groups in this study had lower AST levels than the non-HS-exposed groups. Besides, studies indicate that HS diminishes the activity of antioxidant enzymes and molecules while elevating the serum MDA level (Israr et al. 2021). The addition of BMLP to the diet led to a significant reduction in MDA, another oxidative stress parameter, in both the thermoneutral and HS groups in this study. This suggests that BMLP can effectively neutralize MDA, the end product of lipid peroxidation that arises following oxidative injury.

## Conlusions

Supplementing quail diets with GSP (3%) and BMLP (4%) mitigates the negative impacts of HS on growth performance, egg quality, and serum biochemistry. The addition of GSP and BMLP alleviates the adverse effects of HS on FI and egg quality. In particular, BMLP has shown superiority in preventing egg weight loss. GSP and BMLP also contribute to improved albumen quality and reduced oxidative stress through their antioxidant properties. These findings underscore the potential of natural supplements for enhancing quail resilience to HS and maintaining overall health.

## Data Availability

The data is available from the corresponding author on reasonable request.
